# Subjecting appropriate lung adenocarcinoma samples to next‐generation sequencing‐based molecular testing: challenges and possible solutions

**DOI:** 10.1002/1878-0261.12190

**Published:** 2018-03-23

**Authors:** Weihua Li, Tian Qiu, Yun Ling, Shugeng Gao, Jianming Ying

**Affiliations:** ^1^ Department of Pathology National Cancer Center/Cancer Hospital Chinese Academy of Medical Sciences and Peking Union Medical College Beijing China; ^2^ Department of Thoracic Surgery National Cancer Center/Cancer Hospital Chinese Academy of Medical Sciences and Peking Union Medical College Beijing China

**Keywords:** lung adenocarcinoma, multifocal tumors, next‐generation sequencing, tumor cellularity, tumor heterogeneity

## Abstract

Next‐generation sequencing (NGS) has recently been rapidly adopted in the molecular diagnosis of cancer, but it still faces some obstacles. In this study, 665 lung adenocarcinoma samples (558 TKI‐naive and 107 TKI‐relapsed samples) were interrogated using NGS, and the challenges and possible solutions of subjecting appropriate tissue samples to NGS testing were explored. The results showed that lower frequencies of *HER2*/*BRAF*/*PIK3CA* and acquired *EGFR* T790M mutations were observed in biopsy samples with <20% tumor cellularity than in those with ≥20%, but there were no significant differences in the frequencies of *EGFR* or *KRAS* mutations. Moreover, tumor heterogeneity was assessed by heterogeneity score (HS), which was calculated through multiplying by 2 the mutant allele frequency (MAF) of tumor cells. In TKI‐naive samples, intratumor heterogeneity could occur in *EGFR*,*KRAS*,*HER2*,*BRAF*, and *PIK3CA* mutant tumors, but the degree was variable. Higher *EGFR*, but lower *BRAF* and *PIK3CA* HS values were observed compared with *KRAS* HS. In TKI‐relapsed samples, analysis of concomitant sensitizing *EGFR* and T790M MAFs showed that intratumor heterogeneity was common in acquired *EGFR* T790M mutant tumors. The mutational status between primary and metastatic tumors was usually concordant, but *KRAS*,*HER2*, and *PIK3CA* HS were significantly higher in metastatic tumors than in primary tumors. Additionally, the discordance rate of mutational status in multifocal lung adenocarcinomas diagnosed as equivocal or multiple primary tumors was high. Together, our findings demonstrate that a comprehensive quality assessment is necessary during tissue process to mitigate the challenges of poor tumor cellularity, tumor heterogeneity, and multifocal clonally independent tumors.

**Abbreviations**
CAMS: Chinese Academy of Medical Sciences
FFPE: formalin‐fixed and paraffin‐embedded
HS: heterogeneity score
ISPs: Ion Sphere Particles
MAF: mutant allele frequency
NGS: next‐generation sequencing
NSCLC: non‐small‐cell lung cancer
PGM: Personal Genome Machine

## 1 Introduction

Lung cancer is the leading cause of cancer‐related death in the world (Siegel *et al*., 2017) and can be further divided into small‐cell lung cancer and non‐small‐cell lung cancer (NSCLC). NSCLC accounts for about 85% of lung cancers, and adenocarcinoma is the most common histologic subtype. In these years, patients with advanced lung adenocarcinoma harboring specific genetic alterations have greatly benefited from targeted therapies, as more and more molecular agents are already approved for clinical use or are available from ongoing clinical trials (Mascaux *et al*., 2017).

*EGFR*,* KRAS*,* HER2*,* BRAF*, and *PIK3CA* mutations are these important genetic alterations in the targeted therapies of lung adenocarcinoma. *EGFR* mutations are the most common genetic alterations in lung adenocarcinoma and are more frequent in women, never smokers, and Asian patients (Rosell *et al*., 2009). Patients with sensitizing *EGFR* mutations (exon 19 deletions or L858R mutation) may respond to EGFR‐TKIs treatment (gefitinib, erlotinib, osimertinib, etc.) (Kuan *et al*., 2015; Soria *et al*., 2017), while patients with *EGFR* T790M mutation may benefit from osimertinib (Mok *et al*., 2017). However, *KRAS*,* BRAF*, and *PIK3CA* mutations contribute to resistance to EGFR‐TKIs treatment (Eng *et al*., 2015; Martin *et al*., 2013; Ohashi *et al*., 2012). Recently, dabrafenib plus trametinib has shown robust antitumor activity in NSCLC patients with *BRAF* p.V600E mutation and has been approved by the U.S. Food and Drug Administration (Planchard *et al*., 2016, 2017). Moreover, patients with *HER2* exon 20 insertion mutations may benefit from HER2‐targeted inhibitors, such as afatinib (Mazieres *et al*., 2016). In addition to *EGFR*,* KRAS*,* HER2*,* BRAF*, and *PIK3CA* mutations, mutations in other cancer‐related genes may also act as potentially treatable targets or important prognostic markers (Hyman *et al*., 2017; Lee *et al*., 2017). Therefore, it is critical and necessary to explore the mutation profiling of lung adenocarcinoma accurately and comprehensively to guide further treatment selection.

Next‐generation sequencing (NGS) has been widely used in clinical molecular testing in recent years. Compared to conventional methods, NGS is able to detect multiple genetic alterations in a single assay, with higher sensitivity, fewer amounts of input DNA, shorter time, and lower cost (Ivanov *et al*., 2017). However, there are still many challenges faced in the molecular pathological laboratories, including optimization and familiarization of NGS testing, design and operation of bioinformatics pipeline, and interpretation and reporting of sequence variants. Besides these technical obstacles, challenges related to tumor biological characteristics should also be realized and highlighted. A deep understanding of tumor biology is helpful for the pathologists to select appropriate tissue samples for further NGS‐based molecular testing.

In this retrospective study, somatic mutations of 22 cancer‐related genes in 665 lung adenocarcinoma samples were examined by a validated clinical NGS assay in an ISO15189‐certified laboratory. The challenges related to tumor biological characteristics were explored, and the possible solutions of subjecting suitable tissue samples to NGS testing were discussed.

## 2 Patients and methods

### 2.1 Patients and specimens

Between June 2014 and November 2017, 702 samples were submitted for NGS‐based mutation testing at the Department of Pathology, Cancer Hospital, Chinese Academy of Medical Sciences (CAMS). However, NGS was canceled in 37 samples (37/702, 5.3%), because of scant tissue, less than 10% tumor cellularity, insufficient amount of DNA, or poor quality of DNA. Finally, a total of 665 samples from 661 tumors of 627 patients were enrolled in the study, including 266 resection samples and 399 biopsy samples. All these patients were diagnosed as primary lung adenocarcinoma by pathologists. The study has been approved by the Institute Review Board of the Cancer Hospital, CAMS. The methods were carried out in accordance with the approved guidelines. The informed consents were obtained from all patients.

### 2.2 Tumor cellularity assessment

Tumor cellularity was assessed by the pathologists, as previously described (Li *et al*., 2017). Briefly, the percentage of tumor cells was estimated with 5% increments through the corresponding HE slide 1 and was further corrected through the corresponding HE slide 2. The corresponding HE slide 2 was obtained after the selected block was sectioned to collect enough tumor tissues for DNA extraction. Tumor cell content was assessed by three pathologists independently, and final tumor cellularity was identified through averaging the tumor purity estimated by each pathologist. When macrodissection was used to remove necrosis, mucin lakes, or prominent lymphocytic infiltrates, tumor cellularity was assessed in the selected tumor area for macrodissection.

### 2.3 Genomic DNA extraction

Formalin‐fixed and paraffin‐embedded (FFPE) tissues were collected from the selected blocks and then were subjected to DNA extraction using QIAamp DNA FFPE Tissue Kits (Qiagen, Duesseldorf, Germany), following the manufacturer's instructions. DNA quantity was determined by Qubit 2.0 Fluorometer (Thermo Fisher Scientific, Carlsbad, CA, USA).

### 2.4 Mutation analysis by NGS

The mutational status (including point mutations and indels) of driver genes was tested on the Personal Genome Machine (PGM) platform (Thermo Fisher Scientific), with the Ion AmpliSeq Colon and Lung Cancer Panel. The panel contained 92 pairs of primers targeting 22 cancer‐related genes, including *EGFR*,* KRAS*,* BRAF*,* PIK3CA*,* HER2*,* AKT1*,* NRAS*,* PTEN*,* STK11*,* MAP2K1*,* ALK*,* DDR2*,* CTNNB1*,* MET*,* TP53*,* SMAD4*,* FBXW7*,* NOTCH1*,* ERBB4*,* FGFR1*,* FGFR2*, and *FGFR3*. Briefly, multiplex PCR was performed with 10 ng of genomic DNA, and then, each sample was ligated with unique Ion Xpress Barcodes. After purification and equalization, the amplicon libraries were mixed to prepare the template on Ion Sphere Particles (ISPs), using the Ion OneTouch Template Kit and Ion OneTouch System (Thermo Fisher Scientific). Templated ISPs were loaded onto 316 or 318 chips and then sequenced on PGM. Signal processing, base calling, and alignment were performed using the software of Torrent Suite version 2.0. Variants were annotated by Torrent Variant Caller and further identified with Integrative Genomics Viewer. Mutations were identified when the coverage >1000 and mutant allele frequency (MAF) ≥ 5%.

### 2.5 Calculation of heterogeneity score

The heterogeneity score (HS) values of *EGFR*,* KRAS*,* HER2*,* BRAF*, and *PIK3CA* were calculated as previously described (Li *et al*., 2017). Briefly, assuming that usually one allele was affected by the somatic mutations in tumor cells, the HS was calculated as MAF ×2/tumor cellularity. Therefore, the percentage of tumor cells with a specific somatic mutation could be evaluated by HS. HS < 1 suggested that mutations were present in a subpopulation of tumor cells. HS = 1 suggested that mutations were present in all tumor cells. HS > 1 indicated that copy‐number variation may exist in tumor cells (gain of the mutant allele, acquired uniparental disomy, or loss of the wild‐type allele).

### 2.6 Statistical analysis

The relationships between tumor cellularity, sampling site, and mutation frequencies were investigated by chi‐square test or Fisher's exact test. The differences in HS values among different genes were determined by nonparametric tests (Mann–Whitney *U* and Kruskal–Wallis). The differences between sensitizing *EGFR* and T790M MAFs were compared by correlation analysis and paired Student's *t*‐test. Analysis was conducted using the spss 18.0 software. A two‐sided *P* value < 0.05 was considered statistically significant.

## 3 Results

### 3.1 Patient characteristics

NGS was conducted in 627 patients with lung adenocarcinoma, including 309 male and 318 female patients. Patient ages ranged from 25 to 89 years, with the median age of 60 years. Patient characteristics are listed in Table S1. All tumor samples were further divided into two cohorts, according to whether the patients had received EGFR‐TKIs or not: cohort 1: TKI‐naive samples from patients who had never received EGFR‐TKIs; and cohort 2: TKI‐relapsed samples from patients who had received reversible EGFR‐TKIs (gefitinib, icotinib, or erlotinib) and acquired resistance. There were 558 samples from 554 tumors of 520 patients in cohort 1, including 482 single samples, 28 samples from 14 paired primary and metastatic tumors, eight samples from four tumors (two different blocks from the same tumor), and 40 samples from 20 paired tumors of 20 patients with multifocal lung adenocarcinomas. There were 107 samples from 107 patients in cohort 2, all of which were single samples (Fig. S1).

### 3.2 Mutation profiling

**Figure 1:**
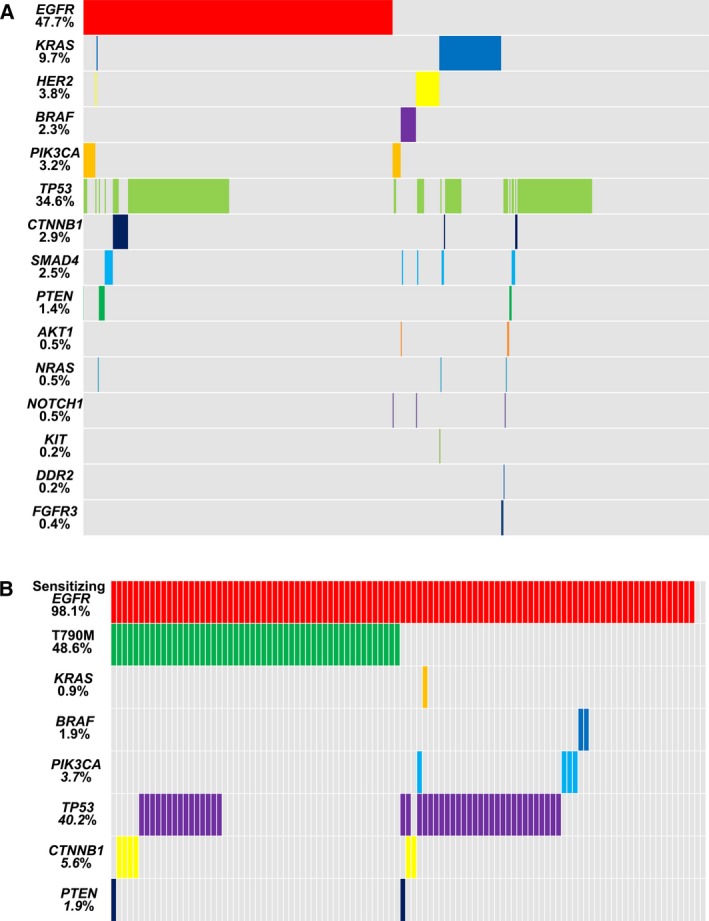
Mutation profiling of patients with advanced lung adenocarcinoma. (A) In cohort 1, a total of 558 samples were tested by NGS, and mutations were observed in 15 genes. (B) In cohort 2, a total of 107 samples were tested by NGS, and mutations were observed in eight genes.

In cohort 1, somatic mutations were observed in 15 cancer‐related genes in 437 of 558 (78.3%) samples. Among the 437 samples, 272 samples had one mutation and 165 samples harbored two or more mutations. The most commonly mutated gene was *EGFR* (266/558, 47.7%). *KRAS*,* HER2*,* BRAF*, and *PIK3CA* mutations were observed in 9.7% (54/558), 3.8% (21/558), 2.3% (13/558), and 3.2% (18/558) of samples, respectively. Other gene mutations (other GMs: any mutation in the remaining 10 genes) were observed in 226 of 558 (40.5%) samples (Fig. 1A). In cohort 2, somatic mutations were observed in eight cancer‐related genes in 105 of 107 (98.1%) samples. Among the 105 samples, 51 samples had one mutation and 54 samples harbored two or more mutations. Sensitizing *EGFR* mutations were observed in 105 of 107 (98.1%) samples, while T790M mutation was observed in 52 of 107 (48.6%) samples. Other GMs (any mutation in the remaining seven genes) were observed in 53 of 107 (49.5%) samples (Fig. 1B).

### 3.3 Tumor cellularity

**Figure 2:**
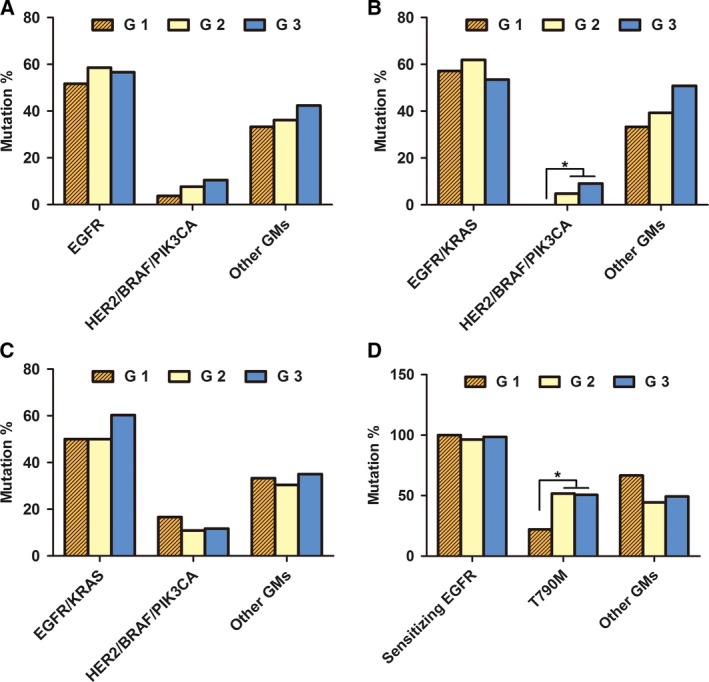
Comparison of mutation frequencies in different tumor cellularity groups. All tumor samples were divided into three groups according to the estimated tumor cellularity (G1: samples with 10–19% tumor cellularity; G2: 20–30% tumor cellularity; and G3: >30% tumor cellularity). In TKI‐naive samples, mutation frequencies of the three groups in (A) biopsy and resection samples, (B) biopsy samples, and (C) resection samples were analyzed. (D) In TKI‐relapsed samples, mutation frequencies of the three groups in rebiopsy samples were analyzed. *P < 0.05.

All samples were divided into three groups according to the estimated tumor cellularity: Group 1 (G1): 10%‐19% tumor cellularity; Group 2 (G2): 20%‐30% tumor cellularity; and Group 3 (G3): >30% tumor cellularity. In cohort 1, there were 27 samples in G1, 130 in G2, and 401 in G3. The results showed that no significant differences in the frequencies of *EGFR*/*KRAS* mutations (mutations in *EGFR* or *KRAS*) and other GMs were observed among G1, G2, and G3. However, the frequency of *HER2*/*BRAF*/*PIK3CA* mutations (mutations in *HER2*,* BRAF*, or *PIK3CA*) in G3 was higher than that in G1, although there were no statistical differences (G3 vs G1, 10.5% vs 3.7%, *P *=* *0.062; G3 vs G2, 10.5% vs 7.7%, *P *=* *0.493; G1 vs G2, 3.7% vs 7.7%, *P *=* *0.224) (Fig. 2A). When only biopsy samples were analyzed, no significant differences in the frequencies of *EGFR*/*KRAS* mutations and other GMs were observed among G1, G2, and G3, whereas lower frequency of *HER2*/*BRAF*/*PIK3CA* mutations was observed in G1 compared with G2 or G3 (G3 vs G1, 9.1% vs 0%, *P *=* *0.006; G2 vs G1, 4.8% vs 0%, *P *=* *0.027; G3 vs G2, 9.1% vs 4.8%, *P *=* *0.228) (Fig. 2B). However, there were no statistical differences among G1, G2, and G3 in resection samples (Fig. 2C). In cohort 2, all samples were rebiopsy samples after resistance to reversible EGFR‐TKIs. There were 9 samples in G1, 27 in G2, and 71 in G3. *EGFR* T790M mutation was more likely to be observed in G2 and G3 compared with G1 (G3 vs G1, 50.7% vs 22.2%, *P *<* *0.001; G2 vs G1, 51.9% vs 22.2%, *P *<* *0.001; G3 vs G2, 50.7% vs 51.9%, *P *=* *0.871.). However, no significant differences in the frequencies of *EGFR* mutations and other GMs were observed among G1, G2, and G3 (Fig. 2D).

### 3.4 Intratumor heterogeneity

**Figure 3:**
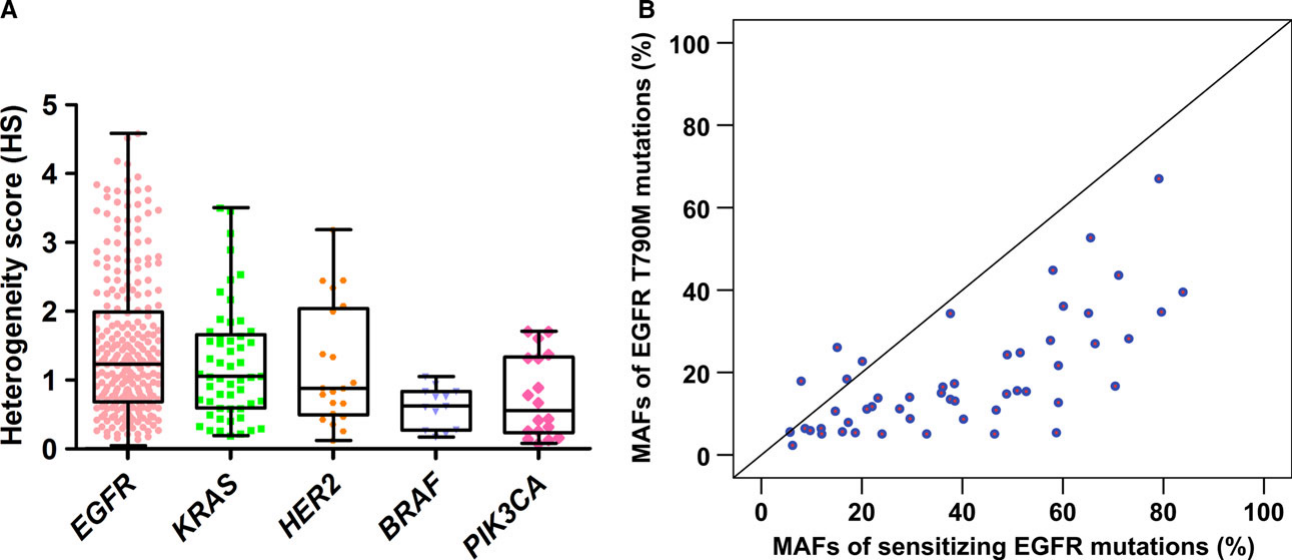
Assessment of intratumor genetic heterogeneity in lung adenocarcinoma samples. (A) Distribution of *EGFR*,*KRAS*,*HER2*,*BRAF*, and *PIK3CA* HS values in cohort 1. (B) Correlation analysis of MAFs in 52 tumors with concurrent sensitizing *EGFR* and T790M mutations in cohort 2.

In cohort 1, the HS values of *EGFR*,* KRAS*,* HER2*,* BRAF*, and *PIK3CA* were calculated to estimate intratumor genetic heterogeneity in lung adenocarcinoma. The results showed that low HS values could be observed in *EGFR*,* KRAS*,* HER2*,* BRAF*, and *PIK3CA* mutant tumors (Fig. 3A). Compared with *KRAS* HS values (median 1.05, IQR 0.59–1.66), significantly higher *EGFR* HS values (median 1.23, IQR 0.68–1.99, *P *=* *0.037) were observed, indicating that mutant allele‐specific imbalance (MASI) may be a common event in *EGFR*. Moreover, *BRAF* (median 0.62, IQR 0.27–0.83, *P *=* *0.022) and *PIK3CA* HS values (median 0.55, IQR 0.23–1.33, *P *=* *0.039) were lower than *KRAS* HS values, indicating that *BRAF* and *PIK3CA* mutations are more likely to be present in a subclonal tumor population. There were no significant differences in HS values between *HER2* (median 0.88, IQR 0.49–2.04) and *KRAS* (*P *=* *0.976). In cohort 2, correlation analysis was performed to assess the MAFs of concurrent sensitizing *EGFR* and T790M mutations. As shown in Fig. 3B, the ratio of T790M/sensitizing *EGFR* in 92.3% (48/52) of tumors was below 100%. By paired Student's t‐test, the results showed that *EGFR* T790M MAFs (mean ± SD, 18.4 ± 13.9) were greatly lower than the concurrent sensitizing *EGFR* MAFs (mean ± SD, 38.7 ± 22.6; *P *<* *0.001).

### 3.5 Comparison of mutations between primary and metastatic tumors

**Figure 4:**
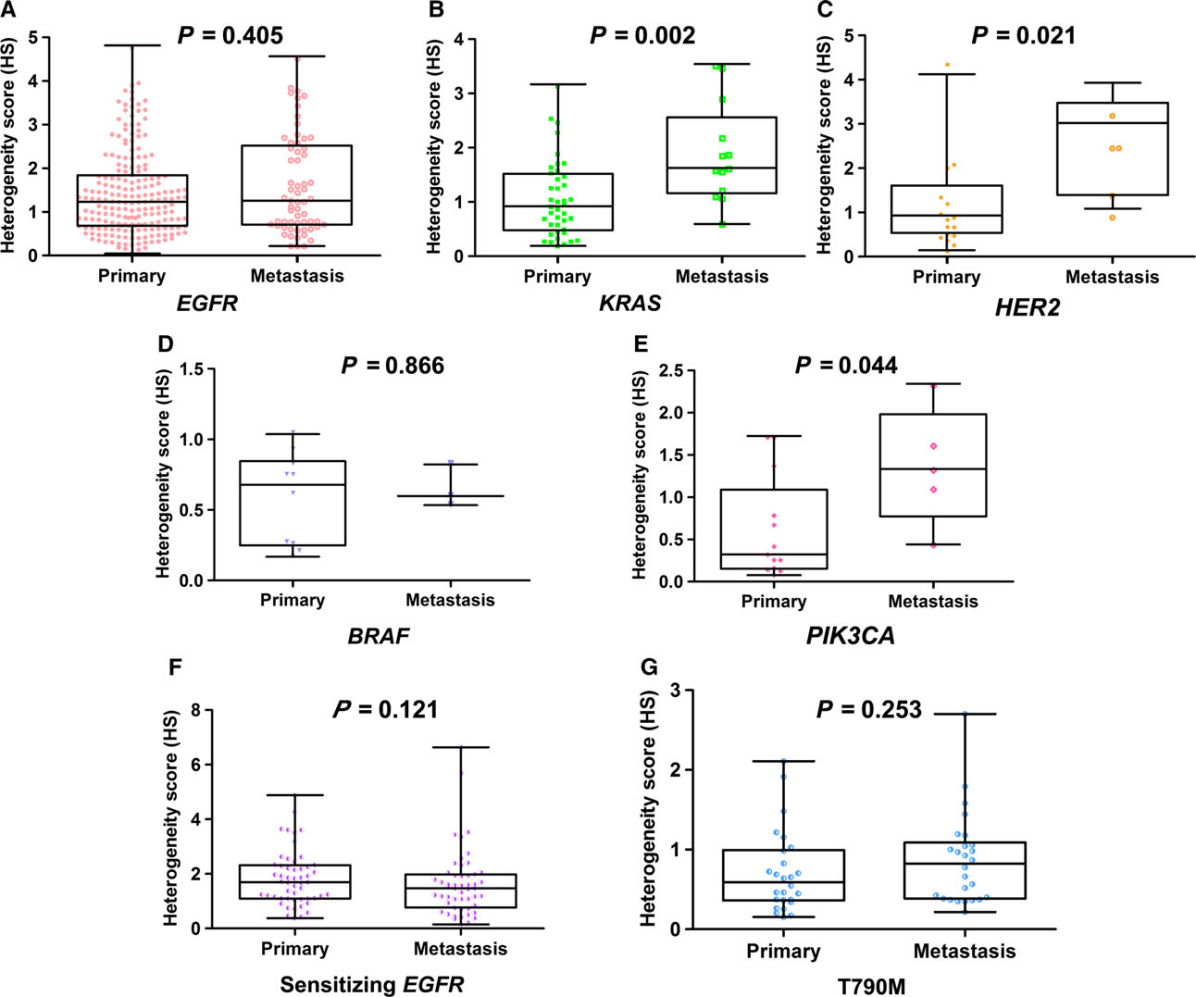
Differences in HS values of driver oncogenes between unpaired primary and metastatic tumors. In cohort 1, (A) *EGFR*, (B) *KRAS*, (C) *HER2*, (D) *BRAF*, and (E) *PIK3CA* HS values were compared between unpaired primary and metastatic tumors. In cohort 2, differences in (F) sensitizing *EGFR* and (G) acquired *EGFR* T790M HS values between unpaired primary and metastatic tumors were examined.

Mutation frequencies between 415 primary tumors and 143 metastatic tumors in cohort 1 were compared. The results showed that no statistical differences in the frequencies of *EGFR*,* KRAS*,* HER2*,* BRAF*, and *PIK3CA* mutations and other GMs were observed between unpaired primary and metastatic tumors (Table 1). In cohort 2, there were also no statistical differences in the frequencies of *EGFR* T790M mutation and other GMs between 54 primary tumors and 53 unpaired metastatic tumors (Table 1). Moreover, mutation profiling of 14 primary tumors and the corresponding metastatic tumors (including nine samples from lymph node, two samples from liver, two samples from brain, and one sample from adrenal gland) was tested in cohort 1. Discordance was observed only in one pair (1/14, 7.7%), in which a *TP53* R273L mutation was present in the primary tumor but not in metastatic lymph node (LN) (Table 2). HS values of *EGFR*,* KRAS*,* HER2*,* BRAF*, and *PIK3CA* mutations between unpaired primary and metastatic tumors were further explored in cohort 1. The results showed that there were no significant differences between unpaired primary and metastatic tumors in the HS values of *EGFR* (primary tumors: median 1.22, IQR 0.68–1.82, *n* = 204; metastatic tumors: median 1.25, IQR 0.70–2.49, *n* = 62; *P *=* *0.405; Fig. 4A) and *BRAF* (primary tumors: median 0.69, IQR 0.25–0.86, *n* = 10; metastatic tumors: median 0.61, *n* = 3; *P *=* *0.866; Fig. 4D). However, the HS values of *KRAS*,* HER2*, and *PIK3CA* mutations were significantly higher in metastatic tumors (*KRAS*: median 1.61, IQR 1.15–2.53, *n* = 13; *HER2*: median 2.45, IQR 1.13–2.81, *n* = 5; *PIK3CA*: median 1.32, IQR 0.76–1.96, *n* = 5) than in primary tumors (*KRAS*: median 0.91, IQR 0.47–1.50, *n* = 41, *P *=* *0.002; Fig. 4B; *HER2*: median 0.75, IQR 0.44–1.30, *n* = 16, *P *=* *0.021; Fig. 4C; *PIK3CA*: median 0.32, IQR 0.15–1.08, *n* = 13, *P *=* *0.044; Fig. 4E). HS values of sensitizing *EGFR* and T790M mutations between the unpaired primary and metastatic tumors were examined in cohort 2, and no significant differences in sensitizing *EGFR* (primary tumors: median 1.70, IQR 1.10–2.32, *n* = 53; metastatic tumors: median 1.47, IQR 0.77–1.98, *n* = 52; *P *=* *0.121; Fig. 4F) and T790M HS values (primary tumors: median 0.59, IQR 0.36–0.99, *n* = 26; metastatic tumors: median 0.82, IQR 0.38–1.09, *n* = 26; *P *=* *0.253; Fig. 4G) between the unpaired primary and metastatic tumors were observed.

**Table 1:** Comparison of mutation frequencies between unpaired primary and metastatic tumors

	Primary tumors (%)	Metastatic tumors (%)	*P*
TKI‐naive samples			
No.	415	143	
*EGFR*	204 (49.2%)	62 (43.4%)	0.231
*KRAS*	41 (9.9%)	13 (9.1%)	0.783
*HER2*	16 (3.9%)	5 (3.5%)	0.846
*BRAF*	10 (2.4%)	3 (2.1%)	0.914
*PIK3CA*	13 (3.1%)	5 (3.5%)	0.951
Other GMs	160 (38.6%)	66 (46.2%)	0.110
TKI‐relapsed samples			
No.	54	53	
Sensitizing *EGFR*	53 (98.1%)	52 (98.1%)	0.989
T790M	26 (48.1%)	26 (49.1%)	0.925
Other GMs	28 (51.9%)	25 (47.2%)	0.628

**Table 2:** Comparison of mutational status in 14 paired primary and metastatic tumors

Case	Location	Tumor cellularity %	Gene	Mutation	Frequency %
55	Lung	90	*EGFR*	p.L747_T751delLREAT	47.6
			*TP53*	p.R273L	13.9
	LN	20	*EGFR*	p.L747_T751delLREAT	8.2
57	Lung	80	*TP53*	p.R248Q	60.0
	LN	60	*TP53*	p.R248Q	45.0
59	Lung	65	*EGFR*	p.E746_A750delELREA	17.7
			*CTNNB1*	p.S45P	17.1
	LN	30	*EGFR*	p.E746_A750delELREA	7.2
			*CTNNB1*	p.S45P	5.0
64	Lung	70	*–*		
	LN	60	*–*		
68	Lung	80	*EGFR*	p.L858R	18.0
			*TP53*	p.R248Q	41.4
	LN	70	*EGFR*	p.L858R	13.8
			*TP53*	p.R248Q	44.9
70	Lung	85	*EGFR*	p.E746_A750delELREA	40.2
			*TP53*	p.R213*	40.3
	LN	40	*EGFR*	p.E746_A750delELREA	12.9
			*TP53*	p.R213*	7.3
71	Lung	80	*EGFR*	p.E746_A750delELREA	7.1
	LN	45	*EGFR*	p.E746_A750delELREA	5.0
82	Lung	80	*–*		
	Brain	30	*–*		
83	Lung	80	*TP53*	p.S241C	33.6
	LN	50	*TP53*	p.S241C	33.5
85	Lung	70	*EGFR*	p.L747_T751delLREAT	46.8
			*TP53*	p.R248G	41.8
	Brain	40	*EGFR*	p.L747_T751delLREAT	33.3
			*TP53*	p.R248G	25.0
89	Lung	60	*TP53*	p.R249S	24.9
	Live	60	*TP53*	p.R249S	38.1
91	Lung	50	*TP53*	p.C242F	7.8
	Adrenal gland	40	*TP53*	p.C242F	12.7
142	Lung	60	*HER2*	p.G776 > VV	26.4
	LN	40	*HER2*	p.G776 > VV	7.1
264	Lung	50	*–*		
	Live	30	*–*		

### 3.6 Mutations in synchronous multifocal lung adenocarcinomas

Twenty paired tumors from 20 patients with synchronous multifocal lung adenocarcinomas in cohort 1 were tested by NGS. The multifocal lung adenocarcinomas occurred in the same lobe or different ipsilateral lobes. Using the criteria reported by Detterbeck *et al*. (2016), matching tumors were diagnosed as multiple primary tumors, intrapulmonary metastasis, and equivocal in seven, seven, and six pairs, respectively. The NGS results showed that discordance of mutational status was observed in eight pairs (8/20, 40%). All cases diagnosed as intrapulmonary metastasis had identical mutations in tumor pairs (7/7, concordance rate 100%). However, only three of seven cases diagnosed as multiple primary tumors had identical mutations in tumor pairs (concordance rate 42.9%), including two ‘wild‐type’ pairs (no mutation was observed in the 22 cancer‐related genes). In addition, two of six cases diagnosed as equivocal had identical mutations in tumor pairs (concordance rate 33.3%), including one ‘wild‐type’ pair (Table 3).

**Table 3:** Comparison of mutational status in synchronous multifocal lung adenocarcinomas

Case	Tumor foci	Tumor cellularity %	Gene	Mutation	Frequency %	Histopathological diagnosis
8	Tumor 1	30	*–*			Equivocal
	Tumor 2	40	*–*			
37	Tumor 1	50	*–*			Equivocal
	Tumor 2	70	*EGFR*	p.L858R	8.9	
50	Tumor 1	40	*TP53*	p.R337L	5.5	Equivocal
	Tumor 2	30	*EGFR*	p.E746_A750delELREA	25.0	
53	Tumor 1	30	*EGFR*	p.L747_P753 > S	16.1	Multiple primary
	Tumor 2	30	*EGFR*	p.L858R	72.8	
56	Tumor 1	80	*EGFR*	p.L858R	71.8	Metastasis
	Tumor 2	30	*EGFR*	p.L858R	51.9	
58	Tumor 1	80	*EGFR*	p.L858R	56.0	Metastasis
			*PIK3CA*	p.H1047Y	6.1	
	Tumor 2	80	*EGFR*	p.L858R	66.3	
			*PIK3CA*	p.H1047Y	5.0	
61	Tumor 1	70	*EGFR*	p.E746_A750delELREA	51.0	Metastasis
	Tumor 2	50	*EGFR*	p.E746_A750delELREA	36.7	
63	Tumor 1	80	*–*			Metastasis
	Tumor 2	40	*–*			
66	Tumor 1	60	*–*			Metastasis
	Tumor 2	60	*–*			
72	Tumor 1	80	*EGFR*	p.L747_S752delLREATS	34.3	Metastasis
	Tumor 2	30	*EGFR*	p.L747_S752delLREATS	23.6	
76	Tumor 1	80	*EGFR*	p.L858R	22.7	Metastasis
	Tumor 2	50	*EGFR*	p.L858R	23.7	
92	Tumor 1	70	*–*			Multiple primary
	Tumor 2	50	*–*			
93	Tumor 1	50	*EGFR*	p.L858R	8.4	Equivocal
	Tumor 2	30	*EGFR*	p.L858R	9.0	
94	Tumor 1	60	*EGFR*	p.E746_A750delELREA	46.9	Multiple primary
	Tumor 2	70	*EGFR*	p.E746_A750delELREA	18.5	
96	Tumor 1	40	*–*			Multiple primary
	Tumor 2	40	*–*			
108	Tumor 1	30	*EGFR*	p.E746_A750delELREA	5.6	Multiple primary
	Tumor 2	40	*KRAS*	p.G12C	9.9	
110	Tumor 1	40	*EGFR*	p.L858R	7.0	Equivocal
			*TP53*	p.Y220*	13.1	
	Tumor 2	30	*–*			
113	Tumor 1	30	*HER2*	p.M774_A775insAYVM	12.5	Multiple primary
	Tumor 2	60	*EGFR*	p.L747_P753 > S	43.4	
122	Tumor 1	40	*KRAS*	p.G12C	5.4	Multiple primary
	Tumor 2	30	*–*			
393	Tumor 1	60	*EGFR*	p.G719A	86.7	Equivocal
	Tumor 2	30	*EGFR*	p.L858R	19.5	

## 4 Discussion

The amplification‐based NGS can detect multiple gene mutations with as little as 10 ng DNA from FFPE samples, with relatively higher performance as compared to conventional methods (Haley *et al*., 2015). Although NGS has been rapidly adopted in molecular diagnosis, there are still some obstacles that should be carefully evaluated during quality control of each step. In this study, a total of 665 lung adenocarcinoma FFPE tumor tissue samples, including 558 resection/biopsy samples from TKI‐naive patients and 107 rebiopsy samples from TKI‐relapsed patients, were tested by the amplification‐based NGS. Challenges posed to the pathologists in how to select appropriate tissue samples were explored. These challenges included poor tumor cellularity, intratumor heterogeneity, heterogeneity between primary and metastatic tumors, and multifocal tumors.

Assessment of tumor cellularity is a necessary process in routine molecular testing. Properly trained and qualified pathologists are required to accurately quantify tumor cell content and to determine whether the minimum tumor cell content is reached. Generally, the minimum tumor cell content is recommended to be more than two times the limit of detection (LOD) in routine mutation testing (Wong *et al*., 2014). Thus, samples with ≥10% tumor cellularity were included in the NGS assay in our laboratory, as the LOD of the NGS platform we used was ~5%. In TKI‐naive samples, no significant differences in the frequencies of *EGFR* or *KRAS* mutations were observed among different tumor cellularity groups. However, lower frequency of *HER2*/*BRAF*/*PIK3CA* mutations was observed in biopsy samples with <20% tumor cellularity as compared to those with ≥20% tumor cellularity. Moreover, the frequency of *EGFR* T790M mutation was greatly lower in TKI‐relapsed samples with <20% tumor cellularity than in samples with ≥20% tumor cellularity, suggesting that 20% tumor purity should be the minimum requirement to identify T790M mutation as the cause of TKI resistance in rebiopsy samples using the amplification‐based NGS testing. Moreover, these data indicate that poor tumor cellularity challenges the accurate molecular detection of lung adenocarcinoma. To minimize the risk of false‐negative results, macrodissection or even microdissection is needed to enrich neoplastic DNA for samples with poor tumor cellularity. In addition, more tissue slides are required to obtain enough number of tumor cells for tiny samples with low tumor cellularity, as neoplastic DNA yield is significantly associated with the number and percentage of tumor cells (Da Cunha Santos *et al*., 2016).

Intratumor heterogeneity may cause hidden and inaccurate mutation testing results, which may have negative impacts on personalized medical care. Studies have investigated the intratumor genetic heterogeneity of various tumors through detecting mutational status (mutation or not) in different regions of tumor samples (Suzuki *et al*., 2017; Zhang *et al*., 2017). However, NGS can provide the information of MAFs, which may indicate intratumor genetic heterogeneity and MASI after normalizing to tumor purity (Dienstmann *et al*., 2017; Li *et al*., 2017). In this study, we evaluated intratumor genetic heterogeneity of lung adenocarcinoma with HS values in TKI‐naive samples. We found that intratumor genetic heterogeneity could be observed in *EGFR*,* KRAS*,* HER2*,* BRAF*, and *PIK3CA* mutant tumors, but the degree was highly variable. The distribution of *KRAS* HS values (median 1.05, IQR 0.59–1.66) in our study corresponded closely to normal distribution of HS values according to the ‘one‐hit hypothesis’ for oncogenes, supporting the notions that *KRAS* mutations are usually clonally dominant truncal mutations and not associated with MASI in lung cancer (Chiosea *et al*., 2011; Uchiyama *et al*., 2003; Yu *et al*., 2017). Compared with *KRAS* HS, significantly lower HS values were observed for *BRAF* and *PIK3C*A. As copy‐number gains in wild‐type alleles are rare in *BRAF* and *PIK3CA* mutations of lung adenocarcinoma (Sasaki *et al*., 2015; Yamamoto *et al*., 2008), these results suggest that *BRAF* and *PIK3CA* mutations are more likely to occur in the subpopulation of tumor cells. However, *EGFR* HS values were higher than *KRAS* HS values, possibly because the concurrence of *EGFR* amplification and mutations occur frequently in patients with lung adenocarcinoma, as we previously described (Shan *et al*., 2015). Moreover, the MAFs of *EGFR* T790M were significantly lower than those of the concurrent sensitizing *EGFR* in most of the TKI‐relapsed samples. These data suggest that intratumor heterogeneity should be taken into account in lung adenocarcinoma, especially when *BRAF*,* PIK3CA*, and *EGFR* T790M mutations are tested. Using bulk tumors or multiregion sampling may be useful to mitigate the challenge of intratumor heterogeneity (Gupta and Somer, 2017). Liquid biopsies, such as circulating tumor DNA (ctDNA) and circulating tumor cells (CTCs), may also be helpful (Pisanic *et al*., 2015; Raimondi *et al*., 2014). However, a lack of sensitivity for detecting low MAFs using NGS may limit the use of liquid biopsies as a good supplement.

Genetic heterogeneity between primary and metastatic tumors has been investigated by several studies, and most of the driver mutations between paired primary and metastatic tumors are reported to be concordant (Goswami *et al*., 2015; Vignot *et al*., 2013). Similarly, our study found that the frequencies of *EGFR*,* KRAS*,* HER2*,* BRAF*, and *PIK3CA* mutations in TKI‐naive samples and the frequencies of sensitizing *EGFR* and T790M mutations in TKI‐relapsed samples showed no statistical differences between unpaired primary and metastatic tumors. Moreover, mutation profiling detected in 14 paired primary and metastatic tumors of TKI‐naive samples showed that 13 of 14 (92.9%) pairs had identical mutational status. However, higher HS values of *KRAS*,* HER2*, and *PIK3CA* were observed in metastatic tumors than in unpaired primary tumors, and heterogeneity between primary and metastatic tumors in copy‐number alterations may partly contribute to the differences (Ferronika *et al*., 2017; Sveen *et al*., 2016). Together, these data indicate that although some genes may be involved in clonal divergence, the use of archived primary tumor in molecular diagnosis is feasible to identify the driver mutations of lung adenocarcinoma. However, low‐MAF events may occur more frequently in NGS testing when the primary tumor tissues are used, to which attention should be paid.

The incident of synchronous multifocal tumors is increasing in lung cancer (Arai *et al*., 2012). The distinguishing of multiple primary tumors from intrapulmonary metastasis is important for accurate staging, but is challenging for the pathologists. Recently, some studies report that the use of NGS appears promising in addressing this challenge, based on the hypothesis that clonally related (intrapulmonary metastasis) and independent tumors (multiple primary tumors) exert different patterns of mutational concordance (Patel *et al*., 2017; Schneider *et al*., 2016). In this study, we found that no discordance of mutational status was detected in all tumor pairs diagnosed as intrapulmonary metastasis by histologic examination, whereas the discordance rate was as high as 61.5% (8/13) in tumor pairs diagnosed as equivocal or multiple primary cancers. Testing the mutational status of all multifocal tumors may provide a guide to diagnosis and to selection of the best treatments. Therefore, it is recommended to subject all multifocal tumors to the NGS‐based molecular testing, especially when equivocal or multiple primary cancers were diagnosed by histologic examination.

There are some limitations in our study. Firstly, only point mutations and indels are explored, and the data of other variants (including copy‐number variants and translocation) are lacked. Secondly, the sample sizes of paired primary and metastatic tumors, as well as tumor pairs from multifocal tumors, are relatively small. Larger sample sizes are needed to further validate the conclusions.

In conclusion, our study demonstrates that ≥20% tumor cellularity is required to identify T790M mutation as the cause of TKI resistance, and to detect *HER2/BRAF*/*PIK3CA* mutations in biopsy samples using the amplification‐based NGS testing. Intratumor heterogeneity can be observed in *EGFR*,* KRAS*,* HER2*,* BRAF*, and *PIK3CA* mutant tumors, but is more likely to occur in TKI‐naive *BRAF*/*PIK3CA* mutant tumors and TKI‐relapsed *EGFR* T790M mutant tumors. Mutational status between primary and metastatic tumors is highly concordant, but *KRAS*,* HER2*, and *PIK3CA* HS values are significantly higher in metastatic tumors than in primary tumors. Moreover, high discordance rate of mutational status may be observed in multifocal lung adenocarcinomas diagnosed as equivocal or multiple primary cancers. Therefore, to achieve optimal NGS testing quality, prospective assessment is critical during tissue process.

## Author contributions

WL, JY, and SG conceived and designed the project and wrote the manuscript. WL and TQ acquired the data; and WL, TQ, and YL analyzed and interpreted the data.

## Supporting information


**Fig. S1.** Lung adenocarcinoma samples subjected to NGS‐base molecular testing.

**Table S1.** Characteristics of 627 lung adenocarcinoma patients.
