# Supplementary figures and images for: Subjecting appropriate lung adenocarcinoma samples to next‐generation sequencing‐based molecular testing: challenges and possible solutions
Source: Mol Oncol. 2018 Mar 23;12(5):677–89. doi: 10.1002/1878-0261.12190 (PMC5928389; doi:10.1002/1878-0261.12190)

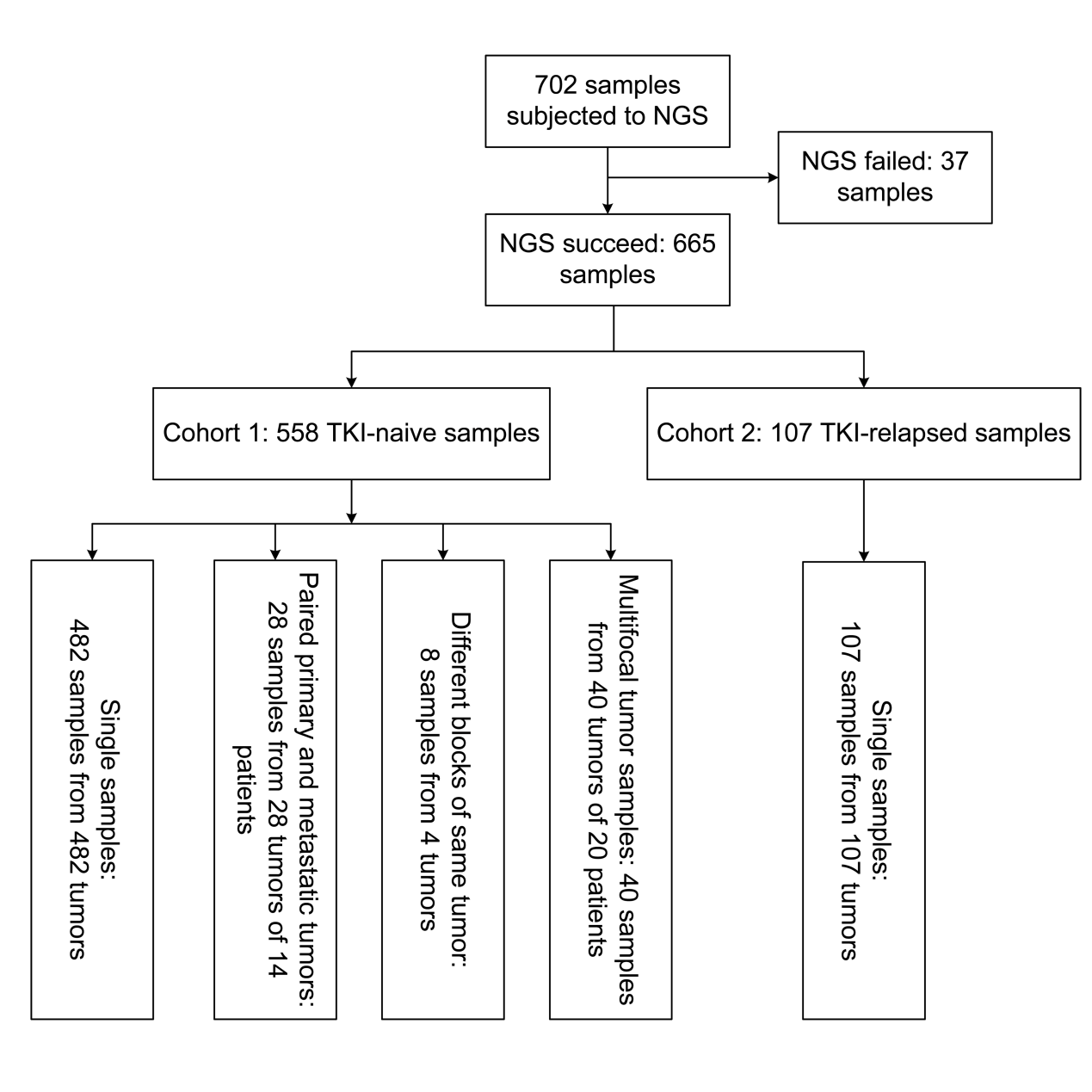


**Supplementary Figure 1** Lung adenocarcinoma samples subjected to NGS-base molecular testing.

Supplement: Supplementary file 1 — Fig. S1. Lung adenocarcinoma samples subjected to NGS‐base molecular testing. [file MOL2-12-677-s001.docx]
